# Optimizing Primary Healthcare in Hong Kong: Strategies for the Successful Integration of Radiology Services

**DOI:** 10.7759/cureus.37022

**Published:** 2023-04-02

**Authors:** Chi Lei Julie Chow, John S Shum, Kei Tat Peter Hui, Andy Fu Chieh Lin, Eric Chun-Pu Chu

**Affiliations:** 1 Proposition Office, EC Healthcare, Hong Kong, HKG; 2 Radiology, Hong Kong Advanced Imaging, Hong Kong, HKG; 3 Radiology, Hong Kong Advanced Imaging Center, Hong Kong, HKG; 4 Chiropractic and Physiotherapy Centre, New York Medical Group, Hong Kong, HKG

**Keywords:** coronavirus, helping people, swimming, public health, radiology, team work and public health, community medicine & public health and radiology, radiology teaching, public health program development

## Abstract

The primary healthcare system in Hong Kong plays a crucial role in addressing the healthcare needs of its population. However, the integration of radiology services into primary care settings has not been fully realized, and there is significant potential for improvement. Incorporating radiology services into primary healthcare can enhance patient care, promote cost-effectiveness, and increase the overall efficiency of the healthcare system by enabling earlier diagnosis and intervention for various health conditions. To successfully integrate radiology services, key strategies include the establishment of public-private partnerships, the adoption of teleradiology and telemedicine services, the development of comprehensive regulatory and policy frameworks, and the exploration of innovative financial models and incentives. By embracing these strategies, Hong Kong can optimize its primary healthcare system and ensure more equitable, effective care for its population.

## Introduction and background

Hong Kong's healthcare system is confronting several challenges, driven by a rapidly growing and aging population, with projections indicating an increase from 7.51 million in 2019 to 8.06 million in 2036 [1]. This growth is primarily due to the expanding elderly population, which is expected to rise by 82%, resulting in 30% of the population being aged 65 or above [1]. Additionally, the increasing prevalence of chronic diseases among both the elderly and middle-aged populations places further strain on healthcare services [1]. Compounding these challenges is service-induced demand, stemming from inefficient coordination and delivery of services such as the overreliance on inpatient care and internal referrals in specialist outpatient cases. As healthcare demand escalates, the system faces persistent issues related to service supply, including manpower shortages, physical space limitations, and funding sustainability. Despite the government's efforts to increase recurrent funding by 45% from $55.6 billion in 2017-18 to $80.7 billion in 2021-22, these challenges will require long-term, innovative solutions to ensure adequate healthcare services for Hong Kong's evolving population [1].

In Hong Kong, primary healthcare (PHC) plays a crucial role as the first point of contact for individuals and families within their living and working communities. Providing accessible, comprehensive, continuous, coordinated, and person-centered care, a well-established PHC system serves as the foundation and portal of the healthcare service pyramid [2]. By managing, maintaining, and enhancing the population's health at the community level, PHC effectively acts as a gateway to specialized secondary and tertiary healthcare in hospital and institution settings [2]. As the most essential component in a well-functioning healthcare system, PHC is integral for ensuring the well-being of Hong Kong's population.

The need for a comprehensive and efficient primary healthcare system is increasingly evident, as Hong Kong faces new challenges resulting from an aging population and rising chronic disease prevalence [2]. To address these issues, a shift in the healthcare focus from curative treatment to disease prevention is imperative. By enhancing district-based PHC services, the goal is to transition the current healthcare system and public mindset from a treatment-oriented approach to a prevention-oriented one. The goals of the Primary Healthcare System are to improve the overall health status of the population, provide accessible and coherent healthcare services, and establish a sustainable healthcare system [2].

Importance of radiology services

Radiology plays an indispensable role in the early detection, diagnosis, and treatment of aging-related and chronic diseases, contributing to better management and improved quality of life for affected individuals [3]. As the population ages, the prevalence of chronic conditions such as cardiovascular diseases, neurodegenerative disorders, cancer, and musculoskeletal conditions rises, necessitating advanced imaging techniques to effectively diagnose and monitor these ailments [4]. Radiological modalities, including X-rays, computed tomography (CT), magnetic resonance imaging (MRI), ultrasound, and nuclear medicine, enable healthcare professionals to non-invasively visualize and assess the extent of disease progression, helping tailor personalized treatment plans [5,6]. Early identification of chronic diseases through radiological screening and diagnostic tests can lead to timely intervention, potentially mitigating disease complications and reducing the burden on healthcare systems [7]. Furthermore, radiology's role in monitoring the efficacy of ongoing treatments empowers clinicians to adjust therapeutic strategies when necessary, ensuring optimal patient outcomes [8]. In summary, radiology's contributions to the early detection, diagnosis, and treatment of aging and chronic diseases are crucial in addressing the challenges posed by an aging population and the escalating prevalence of chronic conditions [4,9].

The growing demand for radiology services in Hong Kong is driven by several factors, including an aging population, an increasing prevalence of chronic diseases, and advancements in medical technology [10]. As the number of elderly individuals continues to rise, so does the need for early detection, diagnosis, and treatment of age-related and chronic conditions such as cardiovascular diseases, cancer, and neurodegenerative disorders [11]. Radiology, with its advanced imaging techniques like X-rays, CT scans, MRI, ultrasound, and nuclear medicine, plays a crucial role in addressing these healthcare challenges [12]. Additionally, the rapid development of medical technology has led to the emergence of more sophisticated and precise imaging modalities, further increasing the demand for radiology services [13]. This growing demand places significant strain on Hong Kong's healthcare system, necessitating investments in infrastructure, equipment, and manpower to ensure that radiology services remain accessible and efficient for the population.

Scope and aim

Integrating radiology services into the primary healthcare system has the potential to significantly improve healthcare service delivery and overall patient care. By providing primary care providers with easy access to diagnostic imaging services, this integration allows for earlier detection and diagnosis of various conditions, including chronic and age-related diseases [14]. This early intervention can lead to more effective treatment plans and better patient outcomes, ultimately reducing the burden on secondary and tertiary healthcare services [15]. Furthermore, the availability of radiology services in the primary care setting promotes interprofessional collaboration between clinicians and radiologists, enhancing communication and enabling more comprehensive patient management [16,17]. The integration of radiology services into the primary healthcare system has the potential to positively impact healthcare service delivery and overall patient care by fostering early detection, timely diagnosis, and efficient collaboration among healthcare professionals.

## Review

Benefits of integrating radiology services into the Primary Healthcare System

Improved Access to Diagnostic Services

Improved access to diagnostic services at the level of primary care in Hong Kong can minimize diagnostic imaging wait times and improve radiological services for patients in distant areas. Integrating radiology services into primary, secondary, and tertiary care facilities can decrease the demand for diagnostic imaging, hence decreasing patient wait times. Improved patient outcomes result from quicker diagnosis and treatment. Improving healthcare access can also be achieved by extending radiology services to primary healthcare centers in remote and underserved regions. Diagnostic imaging closer to patients' homes minimizes travel time and costs, encouraging them to seek care more frequently and earlier [18].

Early Detection and Diagnosis

Incorporating radiology services in primary care settings can significantly contribute to the early detection and diagnosis of various medical conditions, ultimately improving patient outcomes and reducing the healthcare burden. Radiological services, such as X-rays, CT scans, MRI, ultrasound, and nuclear medicine, play a vital role in identifying abnormalities and diseases at their earliest stages, allowing for prompt intervention and treatment [3]. By integrating these advanced imaging techniques into primary care, patients can receive timely diagnoses without the need for referral to secondary or tertiary care facilities, streamlining the diagnostic process.

Programs like stroke screening and cardiac examinations are beneficial to provide early detection and diagnosis in the aging population, which improves patient outcomes and reduces the healthcare system burden by lowering the need for more intrusive and costly procedures later in disease progression.

Better Continuity of Care

Incorporating radiology services into primary care improves continuity of care by encouraging information sharing and healthcare system collaboration and communication. When radiology services are integrated into primary care, primary care practitioners can more easily use diagnostic imaging results to make patient care decisions. This smooth information flow and communication between primary care practitioners and radiologists can lead to a more holistic approach to patient management, ensuring patients receive the most appropriate and timely care. This integration can also improve communication between healthcare personnel, resulting in more coordinated and patient-centered care [19]. As a result, patients can benefit from a cohesive healthcare experience with alternative therapy such as traditional Chinese medicine or chiropractic treatment, with healthcare providers working in unison to address their needs [20]. They can improve continuity of care by facilitating information sharing and strengthening coordination and communication among healthcare providers, ultimately leading to better patient outcomes.

Cost-Effectiveness

Radiology services in primary care can reduce needless referrals to specialists and hospitals and optimize healthcare resource use. Primary healthcare practitioners, including general practitioners, nurses, pharmacists, physiotherapists, and chiropractors, can perform many initial diagnostic examinations with direct access to diagnostic imaging services [21,22]. This technique reduces the number of patients referred to specialists and hospitals for diagnostics, relieving secondary and tertiary care facilities. Specialist consultations and hospital-based imaging services can be reserved for patients with more complex or urgent requirements, which can improve resource allocation. Radiology services in primary care reduce unnecessary specialist visits and hospital stays, saving patients and the healthcare system money. They can reduce wasteful referrals and optimize resource utilization, improving healthcare system efficiency.

Promotion of Preventive Healthcare

The promotion of preventive healthcare is an essential aspect of modern medicine, and radiology plays a significant role in this endeavor, particularly in screening programs for the early detection of chronic diseases [3]. Various imaging techniques, such as mammography, CT scans, and ultrasound, have been proven effective in identifying early signs of conditions like cancer, cardiovascular diseases, and other chronic illnesses [23]. By incorporating radiology services into primary care settings, healthcare providers can encourage patients to participate in regular screening programs and take a proactive approach to their healthcare, ultimately leading to early detection and intervention when necessary [19]. This proactive approach can result in improved patient outcomes, as timely diagnosis and treatment have been shown to be more effective for managing chronic conditions [24]. They play a vital role in promoting preventive healthcare by facilitating screening programs for the early detection of chronic diseases and fostering a proactive approach to healthcare management.

**Figure 1:**
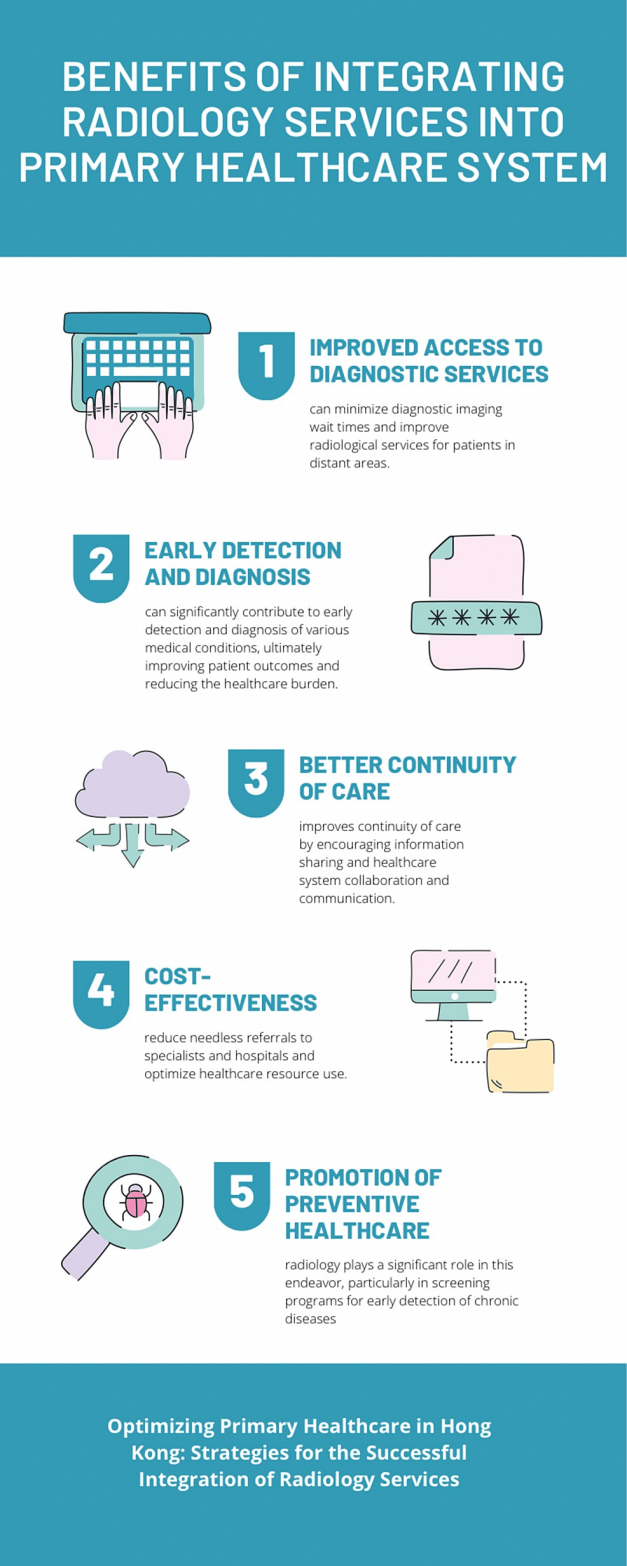
Benefits of Integrating Radiology Services into the Primary Healthcare System

Figure 1 provides a pictorial representation of the benefits of integrating radiology services into the Primary Healthcare System.

Strategies for integrating radiology services in the Primary Healthcare System

Public-Private Partnerships

Public-private partnerships (PPPs) can play a crucial role in establishing radiology facilities within primary healthcare centers by leveraging the strengths of both sectors to improve access to diagnostic imaging services. Through PPPs, the public sector can facilitate the integration of radiology services into primary healthcare by providing the necessary regulatory framework, infrastructure, and support while the private sector can bring in expertise, innovation, and investment in state-of-the-art imaging equipment and technologies [25]. This collaborative approach can ensure the availability of modern diagnostic imaging services at the primary care level, making radiology services more accessible and efficient for patients. Moreover, PPPs can enable the development of sustainable financing models, which can help ensure the long-term viability of radiology facilities in primary healthcare centers [26]. PPP can help primary healthcare centers build radiology services by encouraging public-private collaboration, investing in advanced imaging equipment and technology, and promoting sustainable funding methods.

Tele-radiology and Telemedicine Services

Tele-radiology and telemedicine services have emerged as powerful tools in primary healthcare, leveraging technology to enable remote interpretation of diagnostic images and consultations [27]. Through teleradiology, primary healthcare providers can access radiologists and specialized imaging expertise regardless of their location, ensuring the timely and accurate interpretation of imaging studies [28]. This remote collaboration helps bridge the gap between primary healthcare centers and radiology specialists, potentially resulting in faster diagnosis and treatment. Telemedicine services further complement this approach by facilitating virtual consultations with specialists, enabling primary care physicians to deliver comprehensive, coordinated care for patients in rural and remote areas [29]. By harnessing the power of technology, teleradiology, and telemedicine services can expand access to radiology services and specialist consultations for patients in underserved regions, ultimately promoting greater equity and efficiency in healthcare delivery.

Regulatory and Policy Frameworks

The successful integration of radiology services in primary healthcare settings requires the development and implementation of robust regulatory and policy frameworks to guide this process. Establishing clear guidelines and protocols for the inclusion of radiology services in primary care is crucial for ensuring that patient care is streamlined, effective, and safe [30]. These guidelines should cover imaging study usage criteria, primary care provider certification and training, and primary care physician-radiologist duties. Regulatory frameworks should also require equipment maintenance, radiation safety compliance, and continual quality improvement [31]. Healthcare organizations can integrate radiology services into primary care while maintaining high standards of care and patient safety by building thorough regulatory and policy frameworks.

Financial Models and Incentives

Innovative financial structures and incentives are needed to integrate radiological services into primary care. Public-private partnerships, bundled payment systems, and value-based reimbursement models can help primary care providers afford radiography equipment, staff, and training. Performance-based payment models that promote high-quality, coordinated care, including the proper use of diagnostic imaging services, can also encourage primary care doctors to offer radiology services. Incentives for primary care clinicians to deliver more comprehensive treatment can enhance patient outcomes and healthcare resource use. Innovative financial models and incentive structures can help integrate radiology services into primary care, improving diagnostic imaging access and healthcare delivery.

## Conclusions

Integrating radiology services into Hong Kong's primary healthcare system has numerous benefits. It improves chronic disease management, increases access to diagnostic imaging, and enables proactive care. Public-private partnerships, telemedicine, and investments in technology can help build radiology capacity in primary care centers and rural areas. Comprehensive policies and regulations are necessary to ensure high standards of care and patient safety. Innovative financing models can also facilitate radiology integration, optimizing Hong Kong's healthcare system and improving equity and efficiency. Overall, integrating radiology services into primary care has the potential to enhance healthcare delivery and patient outcomes through improved chronic disease management, patient care, and cost control. Success requires collaboration, research, and continual evaluation from policymakers, healthcare providers, and the community. The innovative financing mechanism will optimize Hong Kong’s healthcare system and improve equity and efficiency. To move this idea ahead, concrete plans and cost-effective analysis would be needed. By working together, these groups can overcome integration challenges, share best practices, and develop novel solutions to advance diagnostic imaging in primary care.
